# Antitumor Effects of Fucoidan Via Apoptotic and Autophagic Induction on HSC-3 Oral Squamous CellCarcinoma

**DOI:** 10.31557/APJCP.2020.21.8.2469

**Published:** 2020-08

**Authors:** Pathanin Chantree, Thanakorn Surarak, Kant Sangpairoj, Panuroot Aguilar, Ekarat Hitakomate

**Affiliations:** 1 *Division of Anatomy, Department of Preclinical Science, Faculty of Medicine, Thammasat University, Pathumthani, 12120, Thailand. *; 2 *Division of Pharmacology, Department of Preclinical Science, Faculty of Medicine, Thammasat University, Pathumthani, 12120, Thailand. *; 3 *Faculty of Dentistry, Thammasat University, Pathumthani, 12120, Thailand. *

**Keywords:** Fucoidan, apoptosis, autophagy, cell cycle, HSC-3 oral squamous cell carcinoma

## Abstract

**Objective:**

Many studies suggested that fucoidan has anticancer potential. The objective of the present study was to determine the cytotoxic effects and mechanism of cell death induced by fucoidan extracted from Fucus vesiculosus on HSC-3 oral squamous cell carcinoma.

**Methods:**

HSC-3 cells were treated with 0, 100, 200, and 400 μg/mL of fucoidan. Cell viability was measured using MTT assay. Apoptosis and cell cycle were measured with a flow cytometry-based assay. Chromatin condensation and nuclear fragmentation were determined using Hoechst 33342 staining. Mitochondrial membrane potential (ΔΨm) was determined using the JC-1 kit. The apoptotic, anti-apoptotic, and autophagic markers study were done by western blot analysis.

**Results:**

the viable cell number of treated HSC-3 cells was decreased. Moreover, treated cells were arrested in the G0/G1 phase. Annexin V/PI staining revealed that fucoidan could induce apoptosis in HSC-3 cells. Western blot analysis suggested the up-regulation of apoptotic markers including cleaved caspase-3, cleaved PARP, Bax, and autophagic markers including LC3-II and Beclin-1 but down-regulation of anti-apoptotic markers, Bcl-2. Fucoidan could disturb ΔΨm and induce chromatin condensation with nuclear fragmentation.

**Conclusion:**

fucoidan has potential in anticancer properties against HSC-3 cells manifested by the induction of apoptosis, cell cycle arrest, and autophagy.

## Introduction

Oral squamous cell carcinoma (OSCC) is one of the cancers that can cause mortality worldwide (Torre et al., 2015). Smoking, tobacco use, alcohol consumption, and HPV infection are the risk factors of oral cavity cancer (Blot et al., 1988; Hashibe et al., 2009). Although treatment methods have developed over the last few decades, the prognosis for patients with OSCC is still moderate or poor. Moreover, the survival rate has only been moderately increased in the past 30 years (Ragin et al., 2007). Standard treatment methods for oral cancer compose of surgery, radiation therapy, or both. Chemotherapy can also be used as an additional treatment however, the controversial outcomes may occur due to its side effects. Only 50% of patients with locally advanced OSCC fail to respond to standard therapies and develop recurrences and distant metastases (Argiris et al., 2008; Leemans et al., 2011). Hence, the development of optional chemotherapeutic agents, especially from natural resources, with more potent but fewer adverse effects is still required.

Fucoidan is a natural sulfated polysaccharide that can be found in the cell wall matrix of brown seaweed. It composes of sulfated L-fucose with small quantities of D-mannose, D-galactose, D-xylose, and uronic acid (Li et al., 2008; Mabeau et al., 1990). Many previous studies have shown that fucoidan enhances antioxidant (Wang et al., 2008), anti-coagulant (Cumashi et al., 2007), anti-inflammatory (Cumashi et al., 2007; Matsumoto et al., 2004), anti-viral (Hayashi et al., 2008), anti-bacterial (Zapopozhets et al., 1995) and immunomodulatory effects (Choi et al., 2005). Fucoidan has also exerted anticancer effects both in vitro and in vivo. Previous in vitro studies suggested that fucoidan down-regulated anti-apoptotic markers in gastric cancer cells (Park et al., 2011), activated caspase cascade in breast cancer cells (Banafa et al., 2013), induced cell death by oxidative stress and disrupted mitochondrial membrane potential in melanoma (Chen et al., 2008). Moreover, a recent study has reported the effect of fucoidan on reducing the invasion ability of OSCC (Lin et al., 2017). Previous in vivo studies reported that fucoidan suppressed the growth of Ehrlich ascites carcinoma (Zhuang et al., 1995), Lewis lung adenocarcinoma (Alekseyenko et al., 2007), 13762 MAT rat mammary adenocarcinoma (Coombe et al., 1987) and reduced the angiogenesis of breast cancer (Xue et al., 2012). However, few studies have determined the antitumor effect of fucoidan in OSCC.

The important characteristic of cancer is apoptotic inhibition (Hanahan and Weinberg, 2011). Therefore, the developing chemotherapeutic agents regarded as potent anticancer ability should have an apoptotic induction effect. Apoptosis is a type of programmed cell death involves in both normal development and diseases. It can be characterized by cell membrane blebbing with cytoplasmic shrinking and nuclear condensation (Burz et al., 2009; Khan et al., 2008). In general, apoptosis can be divided into two pathways including the extrinsic pathway or death receptor pathway and intrinsic pathway or mitochondria-dependent pathway. Both pathways activate caspases and lead to apoptosis. The extrinsic pathway involves the interaction between the ligand and the death receptor whereas the intrinsic pathway involves alteration in mitochondrial integrity by various stimuli (Brenner and Mak, 2009; Jeong and Seol, 2008). Another type of programmed cell death is autophagy. Its mechanism is associated with a self-intracellular degradation system of organelles and proteins in eukaryotic cells (Barth et al., 2010; Galluzzi et al., 2008). Autophagy plays not only as a protective mechanism for cell survival but also as a pro-apoptotic mechanism for cell death in response to external stimuli (Hara et al., 2006). In autophagic processes, the cellular contents are enclosed in autophagosome which is fused with the lysosome to degrade those components (Hamasaki and Yoshimori, 2010). Previous studies have suggested the autophagic induction occurred in cancer due to chemotherapeutic agent treatments (Hamasaki and Yoshimori, 2010; Kondo and Kondo, 2006; Livesey et al., 2009).

In this study, we investigated the antitumor effect with related molecular mechanisms of purified fucoidan extracted from Fucus vesiculosus in HSC-3 oral squamous cell carcinoma. The results provide scientific evidence for the application of fucoidan in oral cancer therapy in the future.

## Materials and Methods

*Fucoidan and cell culture*

95% purified fucoidan from Fucus vesiculosus was obtained from Sigma–Aldrich, St. Louis, MO. The 200 mg/mL of fucoidan stock solution was prepared by dissolving in phosphate-buffered saline (PBS) and stored at −20 °C. HSC-3 oral squamous carcinoma cell line was obtained from Japanese Collection of Research Bioresources Cell Bank. Cells were cultured in DMEM medium supplemented with 1% penicillin-streptomycin (10,000 U/mL) and 10% heat-inactivated fetal bovine serum (Gibco BRL, Gaithersburg, Md., U.S.A.) and maintained at 37°C in an incubator containing 5% CO2.

*Cytotoxicity analysis*

To determine the half-maximal inhibitory concentration (IC_50_) of fucoidan, HSC-3 cells were seeded into 96-well plate for 24 h and then treated with fucoidan diluted in PBS in 0, 10, 50, 100, 150, 200, 250, 300, 350, and 400 µg/ml for 24 h. Thereafter, the MTT working solution (Sigma-Aldrich, USA) was added into each well at a final concentration of 30μg/ml and further incubated for 3 h. OD values were measured by using a spectrophotometer with wavelength at 562 and 630 nm and the results were used to calculate IC_50_ value. Thereafter, HSC-3 cells were treated with three concentrations of fucoidan including the concentration below IC_50_ (100 μg/ml), approximate IC_50_ (200 μg/ml), and above IC_50_ (400 μg/ml) for 24, 48 and 72 h and analyzed cytotoxicity effect by MTT assay. These three concentrations of fucoidan were used for further studies. 

*Annexin V/PI apoptosis assay*

Cells were treated with fucoidan for 48 h. After that, treated cells were analyzed the distribution of apoptotic stages by using FITC Annexin V Apoptosis Detection Kit I (BD Pharmingen™). Briefly, 1 × 10^5^ harvested cells were incubated with FITC Annexin V and PI in the binding buffer and incubate for 15 min at room temperature in the dark. The distribution of apoptotic stages was evaluated by the FACSCalibur flow cytometer (Becton Dickinson, USA).

*Hoechst 33342 staining*

The nuclear condensation and fragmentation were determined by Hoechst 33342 staining (Sigma-Aldrich, USA). After treatment with fucoidan, the culture media were removed and cells were washed in PBS. After that, treated cells were stained with 10 mg/mL Hoechst stock solution diluted at 1:2,000 in PBS and incubated for 15-20 minutes at room temperature in a dark condition. Finally, cells were washed 3 times in PBS and visualized by a fluorescence microscope at 480 nm.

*Determination of mitochondrial membrane potential*

Determination of mitochondrial membrane potential was performed using the JC-1 mitochondria staining Kit (Sigma-Aldrich, USA). Treated HSC-3 cells were incubated with a JC-1 staining solution for 20 min at 37°C with 5% CO_2_. After that, cells were washed twice and overlaid with a growth medium. JC-1 fluorescence intensities of red aggregates and green fluorescence monomers were read with Varioskan LUX Multimode Microplate Reader (Thermo Fisher Scientific Inc., USA). The ratio of the JC-1 aggregates (red fluorescence) versus JC-1 monomer (green fluorescence) for each treatment was evaluated. 

Furthermore, HSC-3 cells (1 × 10^5^) were seeded in 6 well plates for 24 h and were subsequently treated with various concentrations of fucoidan for 48 h at 37°C. Cells were incubated with JC-1 staining liquid for 20 min at 37°C, washed 3 times with growth medium, and examined under the fluorescence microscope.

*Flow cytometry analysis of DNA content*

Treated cells were evaluated cell cycle distribution by using propidium iodide as previously described (Castro et al., 2011). Briefly, after incubated with various concentrations of fucoidan for 48 h, 2 x 10^6^ treated cells were fixed with 70% ethanol and overnight incubated in a -20°C. Cells were washed twice in PBS to remove the ethanol. Cells were then re-suspended in PBS containing 40mg/ml propidium iodide with 100 mg/ml DNAse-free RNAseA (Sigma-Aldrich, USA) and incubated in the dark at room temperature for 15 min. The DNA content was analyzed using the FACSCalibur flow cytometer (Becton Dickinson, USA).

*Western blot analysis*

After treatment with fucoidan, total cellular proteins were obtained using RIPA cell lysis buffer (Cell Signaling Technology, USA) and determined the concentrations by using Lowry protein assays (Bio-rad, USA). From each sample, 30 μg of total proteins were separated by using 15% SDS-PAGE and further transferred onto nitrocellulose membranes. The membranes were blocked in blocking buffer (5% BSA in Tris-buffered saline with 0.1% Tween-20 (TBST)) for 1 h then incubated with primary antibodies (Cell Signaling Technology, USA) including rabbit anti-Bax, rabbit anti-Bcl-2, rabbit anti-cleaved caspase-3, rabbit anti-cleaved PARP, rabbit anti-LC3, rabbit anti-Beclin-1 and rabbit anti-β-actin diluted in 5% BSA in 0.01M TBST at 4°C overnight. The membranes were washed in TBST and further incubated with horseradish peroxidase (HRP) conjugated secondary antibody (Cell Signaling Technology, USA) diluted in 0.01M TBST at 1:5,000 for 1 h at room temperature. The corresponding targeted proteins were visualized by using enhanced chemiluminescent (ECL) detection (Thermo Fisher Scientific Inc., USA). Protei n bands’ intensity was quantified by using ImageJ software.

*Statistical analysis*

Data were expressed as the mean ± SD obtained from triplicate experiments. Statistical analysis was performed using one-way analysis of variance (ANOVA) test provided in SPSS 22. The values obtained in the assays were considered to be statistically significant when P < 0.05.

## Results

*Cytotoxicity effect of fucoidan on HSC-3 cells*

**Figure 1:**
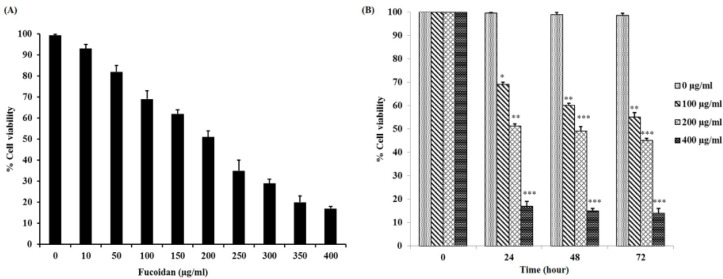
(A) Percent of cell viability of HSC-3 cell line after treatment with various concentrations of fucoidan for 24 h, after that, the IC50 value of fucoidan was calculated. (B) Cytotoxic effect of various concentrations of fucoidan on HSC-3 cells at different time points. Histogram is presented in mean ± SD.*p<0.05, ** p < 0.01, ***p < 0.001

After treatment for 24 h with fucoidan, % cell viabilities of HSC-3 cells were decreased in a concentration-dependent manner from 10 to 400μg/ml compared with the control group (Figure 1A). The 50% inhibitory concentration (IC50) of fucoidan for HSC-3 cells was 201.04 ± 1.02 μg/ml. 

According to IC50, HSC-3 cells were consequently treated with fucoidan (0, 100, 200 and 400μg/ml) for 24, 48 and 72 h. Cell viability was further determined using the MTT assay (Figure 1B). Fucoidan treatment resulted in cell viability reduction in a concentration-dependent manner. The percentage of viability at various concentrations in HSC-3 cells was evaluated as the relative cell viability (%) of viable treated cells compared with viable control cells.

*Analysis of apoptotic cells by flow cytometry in HSC-3 cells*

**Figure 2:**
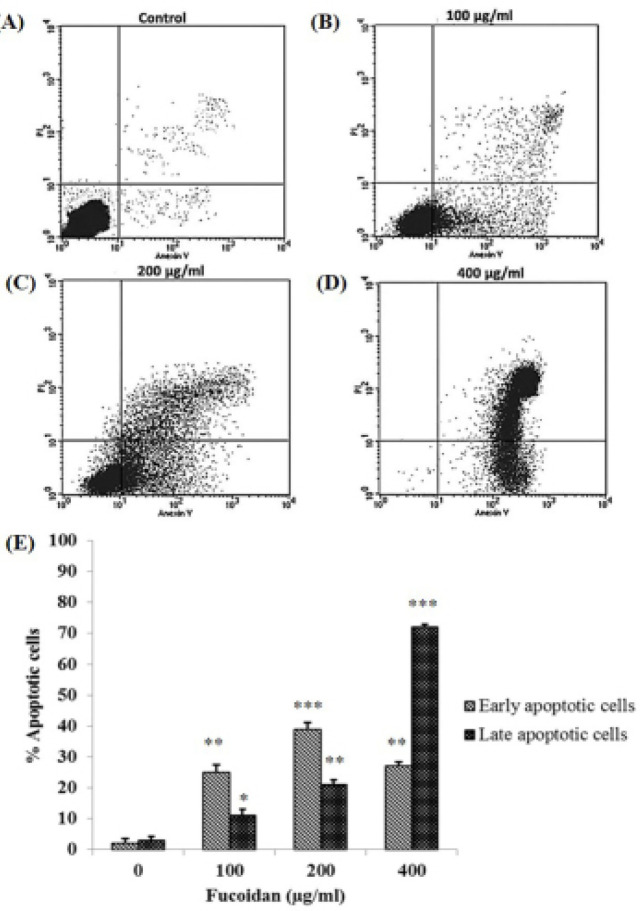
Apoptotic Effects of Fucoidan in HSC-3 Cells were Observed by Using Flow Cytometry. (A-D) represent the results of cells incubated with 0, 100, 200, and 400 μg/ml of fucoidan, respectively. The numbers of AnnexinV-FITC and propidium iodide positive cells are shown in the scattered plot. (E) The percentage of cell populations in early and late apoptotic stages per total cells. *p < 0.05, ** p < 0.01, ***p < 0.001

When apoptosis cells are stained with Annexin V and propidium iodide, the fluorescent dyes can enter the apoptotic cells because of the alteration of the cell membrane. In this study, apoptotic analyses were examined using flow cytometry. The results suggested that several Annexin V positive cells in treated HSC-3 cells were higher than in the control group (Figure 2A, B, C, and D). Moreover, the percentage of treated cells distributed in early and late apoptotic phases were significantly increased after treatment with 100, 200, and 400μg/ml of fucoidan (Figure 2E). The present study confirmed that fucoidan could induce apoptosis in HSC-3 cells. 

*Microscopic observation of HSC-3 cells stained with Hoechst 33342*

**Figure 3:**
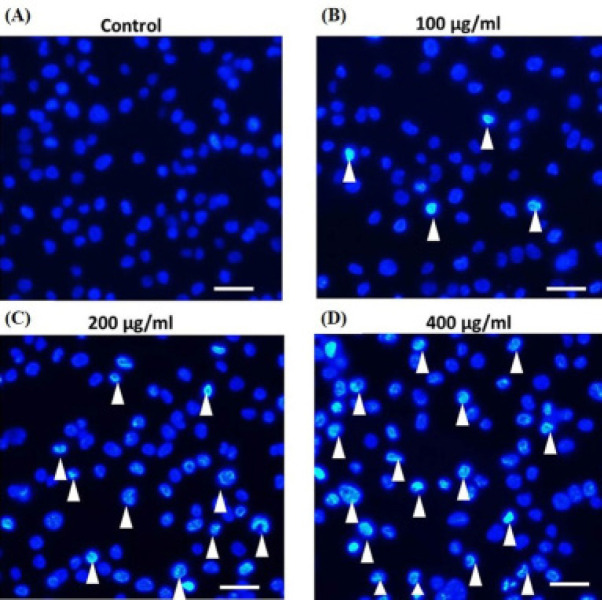
Detection of Nuclear Damages in Treated HSC-3 Cells (A-D) represents the results of cells stained with Hoechst33342 and further observed under a fluorescence microscope after incubated with 0, 100, 200, and 400 μg/ml of fucoidan, respectively. Arrowheads display chromatin condensation and nuclear fragmentation. Scale bar = 50 μm

The morphological changes in the nucleus of apoptotic cells were examined by staining with Hoechst33342. After treatment with fucoidan for 48 h, the characteristics of nuclear damage in treated cells including bright bluish fluorescence nuclei with chromatin condensation and nuclear fragmentation were observed (Figure 3B, C, and D) meanwhile the control cells exhibited nuclei with uniform staining (Figure 3A). Because of the results in nuclear alteration effects, it suggested that fucoidan could induce apoptosis in HSC-3 cells.

*Determination of mitochondrial membrane potential stained with JC-1*

**Figure 4:**
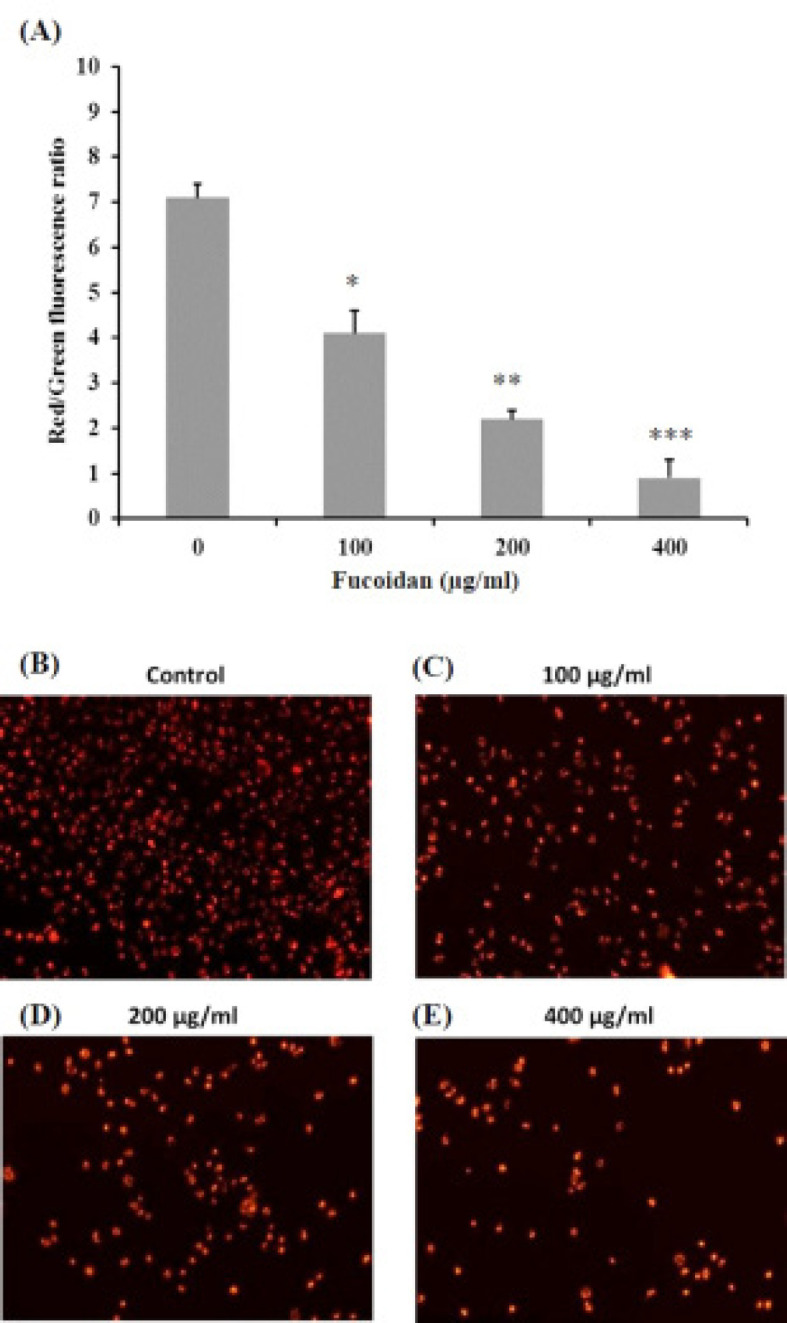
Effects of Fucoidan on Mitochondrial Membrane Potential and Apoptosis in Cultured HSC-3 Cells. (A) The red to green fluorescence intensity of JC-1 ratio was significantly decreased in the concentration-dependent manner (* p < 0.05, ** p < 0.01, ***p < 0.001) compared with the control group. (B-E) The observation under the fluorescence microscope revealed the decreased number of HSC-3 cells with red fluorescence in a concentration-dependent manner from 0-400 μg/ml of fucoidan. Scale bar = 50 μm

The alteration of mitochondrial membrane potential (ΔΨm) is one of the indicators of apoptosis. The effect of fucoidan on ΔΨm of HSC-3 cells was determined by using JC-1 dye. In healthy cells, red fluorescence implies the JC-1 dye forms aggregates in intact mitochondria. In apoptotic cells, the presentation of green fluorescence refers to the monomer of JC-1 dye in mitochondria with low ΔΨm. Therefore, mitochondrial depolarization is also illustrated by a reduction in the intensity ratio of red to green fluorescence. Applications of increasing concentrations of fucoidan caused a significant reduction in the JC-1 ratio (Figure 4A) and the observation of fucoidan treated cells under fluorescence microscope revealed the decreased number of HSC-3 cells with red fluorescence in a concentration-dependent manner (Figure 4B, C, D, and E). These results suggested that fucoidan depolarized the mitochondrial membrane potential and induced apoptosis in HSC-3 cells.

*Effect of fucoidan on cell cycle distribution of HSC-3 cells*

**Figure 5:**
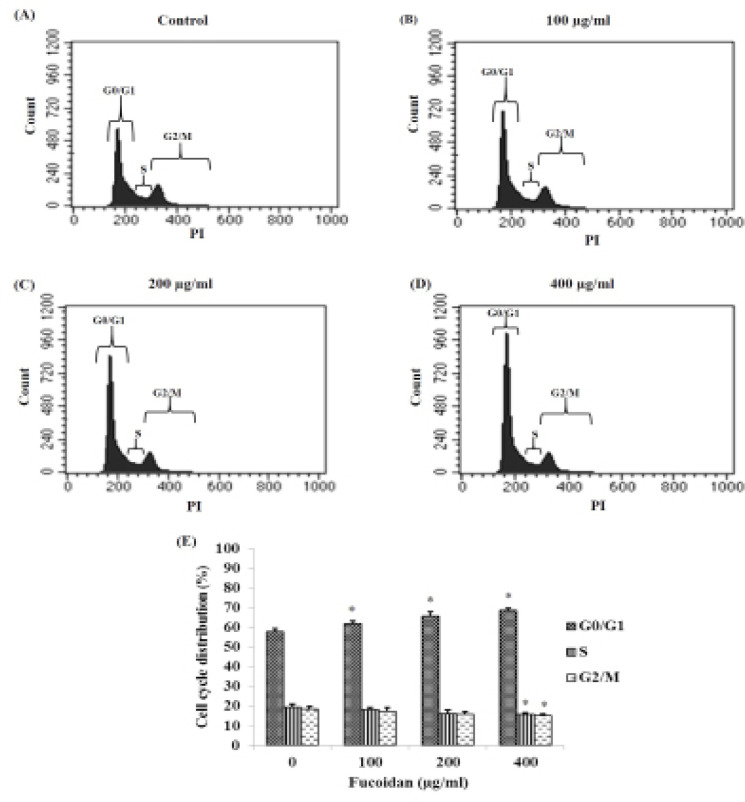
Cell Cycle Analyses of HSC-3 Cells Treated with Fucoidan were Observed by Using Flow Cytometry. The results suggested the distributions in various phases of the cell cycle of HSC-3 cells treated with various concentrations of fucoidan (A): control group; (B), (C) and (D): Fucoidan-treated groups at 100, 200, and 400 μg/ml, respectively. (E) Percentage of each phase are presented as mean ± SD.* p < 0.05

After HSC-3 cells were treated with various concentrations of fucoidan, cells were stained with PI and the distribution of each phase in the cell cycle was determined using flow cytometry. The results revealed that fucoidan could induce an arrest of HSC-3 cells in the G0/G1 phase (Figure 5A, B, C, and D). In this phase, the number of arrested cells was significantly increased in a concentration-dependent manner (Figure 5E). However, the ratios of cells in S and G2/M phase were significantly decreased only in cells treated with high dose (400μg/ml) of fucoidan when compared to control group (Figure 5E)

*Expression analyses of apoptotic related proteins by western blot*

**Figure 6:**
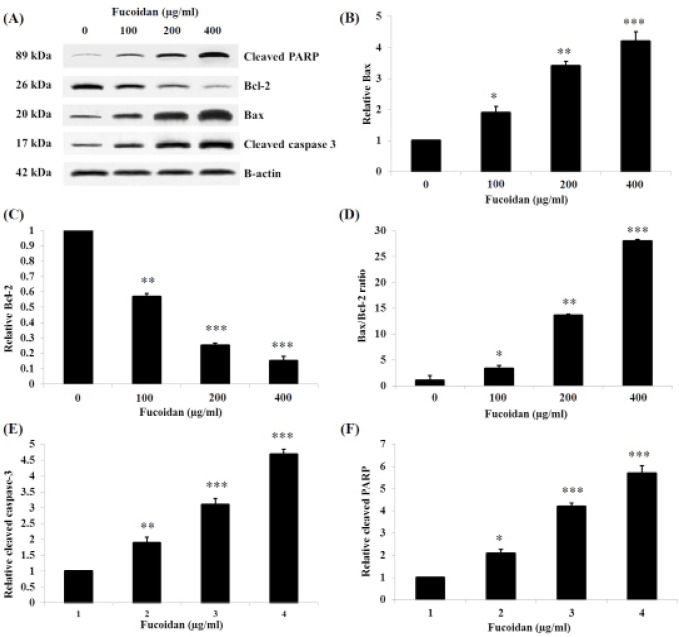
Western Blot Analysis of the Expression Levels of Apoptotic Related Proteins in HSC-3 Cells Treated with 0, 100, 200, and 400 μg/ml of Fucoidan for 48 h. (A) western blot analysis of Bax, Bcl-2, cleaved caspase-3, and cleaved PARP protein expressions. Relative expression levels of (B) Bax, (C) Bcl-2, (D) The ratio of Bax and Bcl-2, (E) cleaved caspase-3, and (F) cleaved PARP were analyzed and compared with the control group. * p< 0.05, ** p < 0.01, *** p < 0.001

Apoptotic effects at the protein expression level of fucoidan in HSC-3 cells were also evaluated. After treatment with fucoidan for 48 h, the expression levels of apoptotic and anti-apoptotic markers were observed (Figure 6). For apoptotic markers, Bax (Figures 6A and B) and cleaved caspase-3 (Figures 6A and E), were significantly increased in a concentration-dependent manner. Conversely, the expression of Bcl-2, an anti-apoptotic marker, was significantly decreased (Figures 6A, 6C). Also, the ratio of Bax to Bcl-2 was significantly increased following the treatment (Figure 6D). Besides, the relative expression level of cleaved PARP, one of the apoptotic markers, was significantly increased in a concentration-dependent manner (Figures 6A and F).

*Morphological and biochemical evidence of autophagy induced by fucoidan*

**Figure 7:**
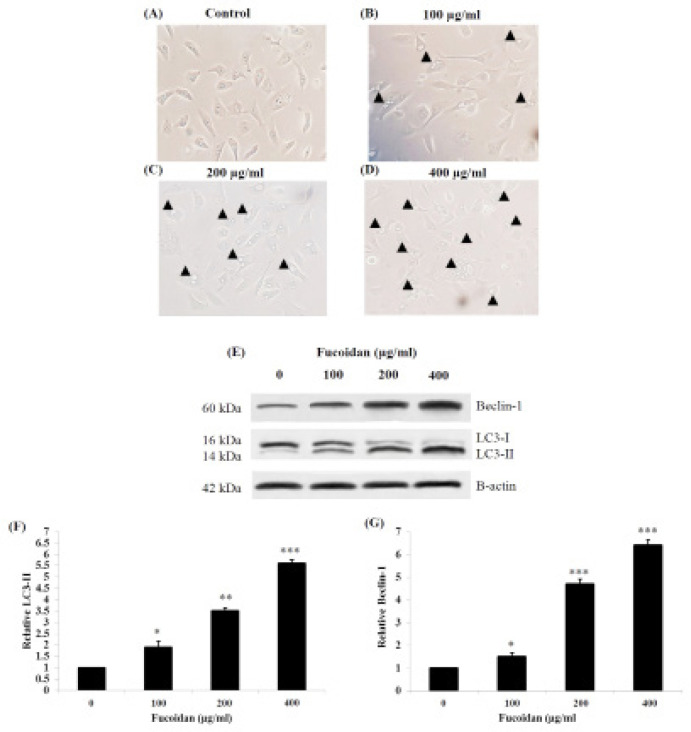
Morphological Changes of Treated HSC-3 Cells were Observed Using a Phase-Contrast Microscope. (A) In control cells, a small number of vacuoles and granules (arrowheads) in the cytoplasm were observed. However, numerous vacuoles and granules were observed in the cytoplasm and cell surface of treated cells (B-D), and the quantities of those changes were increased in a concentration-dependent manner. (E) The levels of LC3-II and Beclin-1 expression were determined by using western blot. Relative expression levels of LC3-II (F) and Beclin-1 (G) were analyzed and compared with the control group. * p < 0.05, ** p < 0.01, *** p < 0.001. Scale bar = 50 μm

After fucoidan treatment, several granules and vacuoles appeared in the cell surface and cytoplasm of HSC-3 cells. The amounts of these morphological changes were developed in a concentration-dependent manner (Figures 7A, B, C, and D). Furthermore, to evaluate whether granular and vacuole appearances were involved with autophagy, LC3-II, and Beclin-1 which are the protein markers for autophagy were examined. In this study, the results suggested that the levels of LC3-II (Figures 7E and F) and Beclin-1 (Figures 7E and G) were significantly increased in a concentration-dependent manner following the treatment. These results implied that fucoidan could induce autophagy in HSC-3 cells.

## Discussion

Many previous studies suggested that fucoidan, a sulfated polysaccharide extracted from brown algae, exerts the suppression of growth and proliferation of human cancer cells through apoptotic mechanisms (Atashrazm et al., 2016; Blaszczak et al., 2018; Han et al., 2015; Kim et al., 2010; Narayani et al., 2019; Park et al., 2011). Moreover, one study reported the effect of fucoidan in reducing the invasion of oral squamous cell carcinoma CAL27 cell line (Lin et al., 2017). Hence, this present study aimed to determine the effect of fucoidan in cytotoxicity induction and identify the related biochemical mechanisms on cultured HSC-3 oral squamous cell carcinoma. 

In the present study, it was revealed that fucoidan reduced cell viability by apoptotic induction. The number of Annexin V positive cells with nuclear chromatin condensation and DNA fragmentation was increased after treatment with fucoidan in a concentration-dependent manner. Apoptosis is a programmed cell death induced by signaling cascades in the intrinsic and extrinsic pathways that finally involve activation of the same target caspases and apoptotic molecules (Brenner and Mak, 2009; Jeong and Seol, 2008). It has been demonstrated that Bax induces apoptosis via mitochondrial outer membrane permeabilization then results in releasing cytochrome c from the intermembrane space of mitochondria to the cytoplasm that initiates caspase-activating pathways. Conversely, Bcl-2 inhibits translocation of cytochrome c then results in inhibition of the caspase activation cascades in the intrinsic apoptotic pathway (Brenner and Mak, 2009; Jeong and Seol, 2008). The present study revealed that fucoidan could induce apoptosis in HSC-3 cells as shown in the decrease of Bcl-2 but increase of Bax levels. Therefore, these expressions indicated that fucoidan increased Bax /Bcl-2 ratio in HSC-3 cells. Previous studies suggested that mitochondrial dysfunction in the intrinsic apoptotic pathway is associated with an increase in the Bax /Bcl-2 ratio (Mackey et al., 1998).

The caspase family proteins are produced as precursor molecules. After they are cleaved at specific sites, the active forms of these molecules act as mediators of apoptosis in both extrinsic and intrinsic pathways (Stennicke and Salvesen, 1998). In the extrinsic pathway, the ligands bind to the death receptors on the cell membrane and stimulate caspase-8. This caspase cleaves Bid which is a molecule that promotes caspase cascades. In the intrinsic pathway, alterations in the mitochondrial membrane potential due to various stimuli result in releasing cytochrome c to the cytosol that further activates caspase-9 (Brenner and Mak, 2009). Activated caspases including caspase-8 and -9 can activate effector caspases including caspase-3 and caspase-7 which cleave various molecules such as Poly (ADP-ribose) polymerase or PARP. PARP is the anti-apoptotic protein according to its DNA repairability, but when it is cleaved, it serves as a marker of apoptosis (Jin and El-Deiry, 2005). Our data revealed that fucoidan up-regulated active caspase-3 and cleaved form of PARP expression in a dose-dependent manner. 

For mitochondria, interruption of mitochondrial membrane potential (ΔΨm) by increasing the permeability of the outer mitochondrial membrane can lead the cell to apoptosis (Green and Reed, 1998; Philchenkov, 2004). Previous studies suggested that fucoidan induced apoptosis in cancer cells by ΔΨm alteration (Kim et al., 2010; Narayani et al., 2019; Park et al., 2011). The present study revealed that fucoidan induced apoptosis involved a decrease in the number of cells with intact ΔΨm determined by fluorescent microscope and optical density measurement of JC-1 staining. Therefore, according to the effects of fucoidan in the up-regulation of Bax, cleaved caspase-3, and cleaved PARP, down-regulation of Bcl-2 and alteration of ΔΨm, it could be implied that fucoidan has potential in apoptotic induction via an intrinsic pathway on HSC-3 oral squamous cell carcinoma. 

Uncontrolled division with a high rate is the distinct feature of cancer. The mechanism behind this characteristic is associated with mutation of cell cycle regulatory genes which allows for a cell to avoid the checkpoint control mechanisms (Hanahan and Weinberg, 2011). In the present study, we found that treatment with fucoidan in HSC-3 cells could exert cell cycle arrest in the G0/G1 phase. However, in the previous study, arrest in S/G2 phase was observed in head and neck squamous cell carcinoma H103 and FaDu cell lines treated with fucoidan (Blaszczak et al., 2018). It has been reported that fucoidan treatment in various cell lines could induce cell cycle arrest in various phases. Cell cycle arrest in the G1 phase was observed in the acute promyelocytic leukemia cell line incubated up to 48 h with fucoidan extracted from Fucus vesiculosus (Atashrazm et al., 2016). The same trend was also observed in MCF-7 breast cancer cells and HT29 colorectal cancer cells (Banafa et al., 2013; Han et al., 2015). However, in MCF-7 breast cancer cells, a combination of fucoidan with other chemotherapeutic agents also increased the number of cells arrested in the G2/M phase (Zhang et al., 2013). We hypothesized that fucoidan induced cell cycle arrest in the G1 phase in HSC-3 cells may be associated with down-regulation of cyclin D1 and CDK-4 but up-regulation of p21WAF1 as suggested by previous studies (Banafa et al., 2013; Han et al., 2015)

Autophagy and apoptosis may occur together as illustrated by the degradation of depolarized mitochondria in autophagosomes (Kondo and Kondo, 2006; Livesey et al., 2009). The previous study suggested the antitumor effect of fucoidan via autophagic induction on AGS human gastric cancer cells (Park et al., 2011). Our results revealed that after treatment with fucoidan, the cytoplasm of HSC-3 cells developed suspected granules and vacuoles. Moreover, the number of cells that presented with these structures was directly related to a concentration of fucoidan. To confirm the involvement with autophagy, we examined the effects of fucoidan treatment on the expression of autophagic markers including LC3-II and Beclin-1. In autophagic mechanisms, the LC3-I protein in the cytoplasm is bound to phosphatidylethanolamine and converted to lapidated LC3-II protein which is presented in the membrane of autophagosome (Asanuma et al., 2003). For Beclin-1, it is related to phosphoinositide-3-kinase and also associated with the membrane nucleation of autophagosome (Jin and White, 2007). As shown in western blot analysis results, the LC3-II was increased in a concentration-dependent manner of fucoidan treatment whereas, in the same concentration, the LC3-I was gradually decreased. These results implied the conversion of LC3-I to LC3-II. In the same trend with LC3-II expression, the level of Beclin-1 levels was significantly up-regulated in a concentration-dependent manner when compared to the control. These results suggested that the antitumor effects of fucoidan are also involved with autophagic induction.

In conclusion, all results presented in this study revealed that fucoidan could suppress the growth and proliferation of HSC-3 oral squamous cell carcinoma via the induction of apoptosis, autophagy, and cell cycle arrest. These results provide the mechanisms associated with the antitumor activity of fucoidan that may be used as an effective therapeutic agent against oral squamous cell carcinoma in the future.
